# A Rare Case of Moyamoya Disease in a Patient With Sickle Cell Disease

**DOI:** 10.7759/cureus.40730

**Published:** 2023-06-21

**Authors:** Ayman Mohamed, Samuel Raterink

**Affiliations:** 1 Internal Medicine, Ascension St. John Hospital, Detroit, USA

**Keywords:** internal carotid artery stenosis, sickle cell disease, stroke-like symptoms, transient ischemic attack, moyamoya disease (mmd)

## Abstract

Moyamoya disease (MMD) is an infrequent progressive cerebral pathology that primarily affects the branches of the internal carotid artery, resulting in stenosis of the internal carotid artery and subsequent development of multiple collateral vessels. As the disease advances, it manifests through various clinical presentations, including stroke and seizures. Prevalence rates indicate a higher incidence among individuals of Eastern Asian descent, while it is notably less common among African Americans. This case report describes the clinical presentation of a 32-year-old African-American female with a past medical history of sickle cell disease and stroke, who sought medical attention at our institution due to a deterioration in left-sided weakness and left wrist drop. Although the patient had been diagnosed with MMD at an early age, no previous medical records were available. Diagnostic evaluation utilizing brain imaging techniques confirmed the presence of MMD, exhibiting minute collaterals that had replaced the middle cerebral artery.

## Introduction

Moyamoya disease (MMD) is an uncommon and progressive intracranial pathology [1]. It is typically associated with stroke or stroke-like symptoms, often accompanied by seizure disorders, primarily observed in children. The incidence of MMD varies among different populations, with African Americans exhibiting notably low occurrence rates [2,3]. However, it has been reported as a potential complication of sickle cell disease [4]. This case report highlights the clinical presentation of a 32-year-old female patient with a history of sickle cell disease and previous strokes. The patient sought medical attention due to worsening weakness from baseline, prompting a comprehensive evaluation. Subsequent imaging studies were performed, leading to the confirmation of the diagnosis of MMD.

## Case presentation

The patient is a 32-year-old female with a medical history of sickle cell disease and childhood seizures. At the age of 4, the patient suffered a stroke that left her with residual left upper extremity weakness due to a diagnosis of MMD; however, medical records pertaining to the incident were not obtainable. The patient presented to our healthcare facility to undergo an evaluation for acute onset of worsening left-sided weakness and numbness. The onset of symptoms began abruptly 10 hours before her hospital presentation with numbness in her left upper extremity. The numbness progressed in severity and was accompanied by increasing weakness and left wrist drop. The patient denied any other accompanying symptoms at the time of presentation. She follows up regularly with her hematologist as an outpatient and has been on long-term hydroxyurea and folic acid therapy. Additionally, the patient undergoes monthly exchange transfusions.

**Figure 1:**
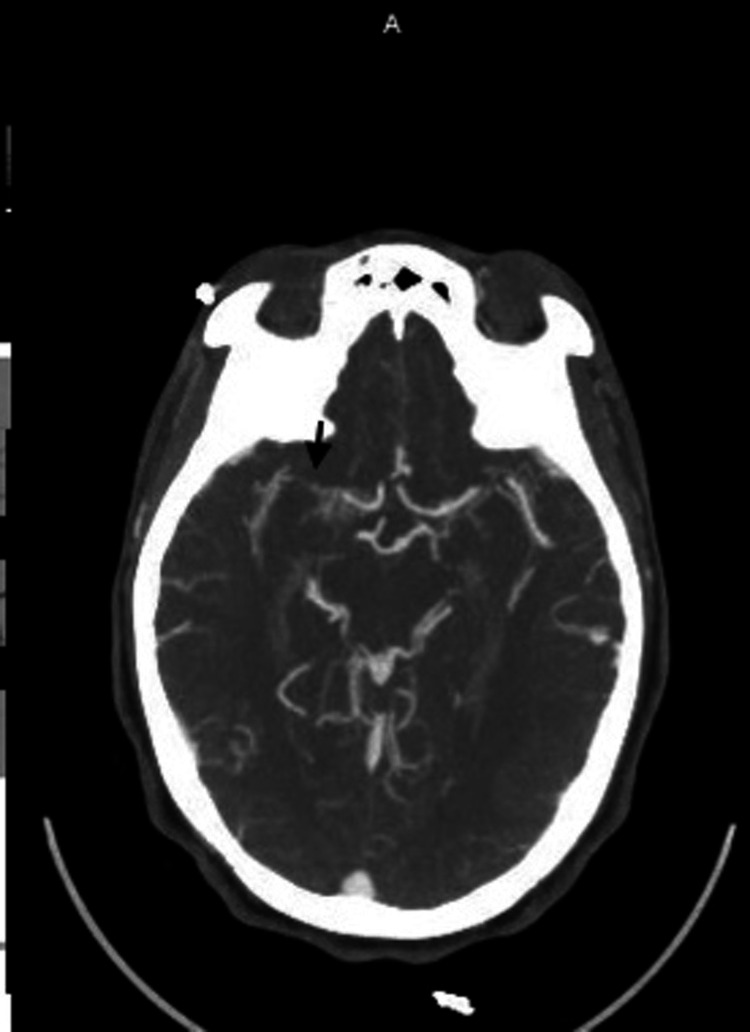
Computed tomography angiography of the head showing stenotic lesion within the horizontal segment of the right MCA MCA, middle cerebral artery

**Figure 2:**
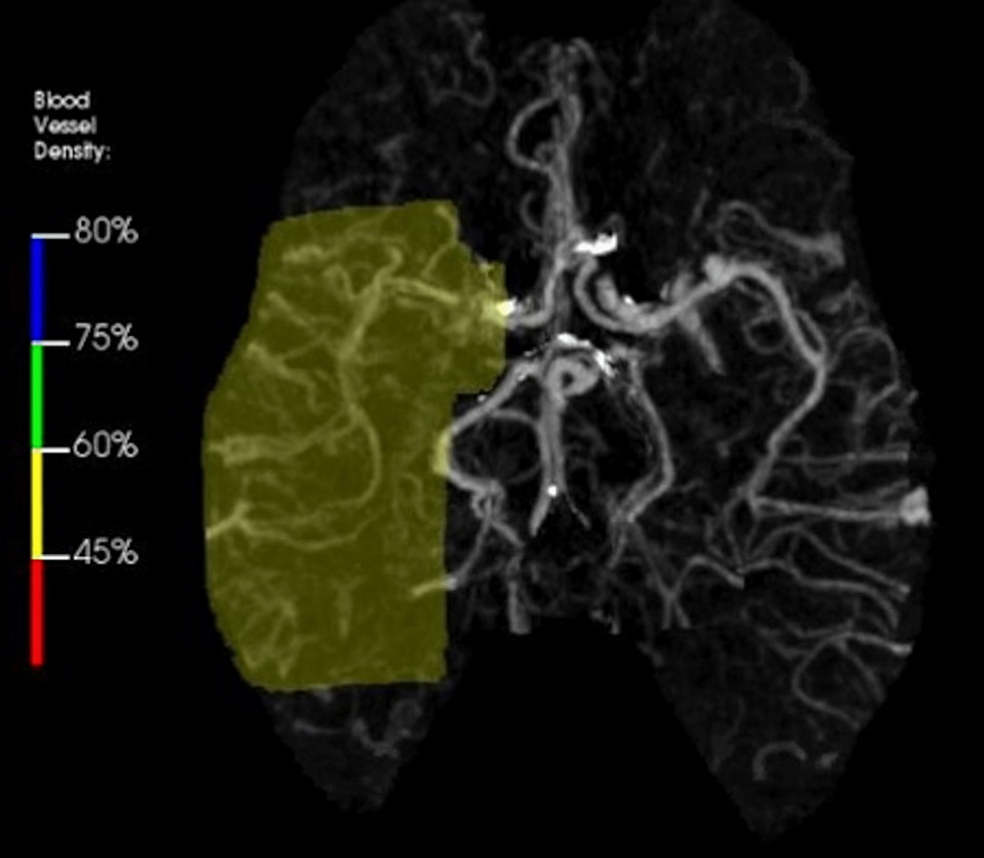
Decreased blood vessel density in the distribution of the right MCA on the rapid color map MCA, middle cerebral artery

Upon presentation to the emergency department, the patient’s vital signs did not exhibit any significant abnormalities. However, physical examination revealed left upper extremity weakness with a strength of 3 out of 5, indicating a decrease in strength compared to their usual 4/5 baseline, and left wrist drop, as well as decreased sensation on the affected side. The remaining physical examination findings were unremarkable. The laboratory results indicated a hemoglobin level of 10 g/dL, a platelet count of 478 K/mcL, a total cholesterol level of 142 mg/dL, and an LDL level of 83 mg/dL, without any other notable abnormalities. A computed tomography scan of the head without contrast exhibited no indications of intracranial hemorrhage, midline shift, or space-occupying lesions. The computed tomography angiography of the head and neck exhibited a stenotic lesion in the horizontal segment of the right middle cerebral artery (MCA) (Figure 1), along with decreased blood vessel density in the distribution of the right MCA on the rapid color map perfusion blood vessel density (Figure 2). However, the computed tomography perfusion study showed no abnormalities in cerebral blood flow. Furthermore, magnetic resonance imaging of the brain without contrast did not exhibit any signs of acute ischemia or infarction.

Since obtaining prior medical records confirming the patient’s diagnosis of MMD proved to be challenging, a diagnostic cerebral angiogram was performed. The results of the procedure indicated the absence of any intracranial aneurysms, but it did show a tangle of minute collaterals replacing the right MCA consistent with MMD (Figure 3). No surgical intervention was deemed necessary at this point, and the patient was instead managed medically through the administration of aspirin 81 mg once daily and atorvastatin 40 mg once daily. Following her admission to the hospital and the initiation of medical management, the patient’s symptoms gradually improved over the next six days, with a return of her left upper extremity weakness to her baseline prior to the onset of her new symptoms.

## Discussion

MMD is an intracranial pathology that exhibits progressive stenosis of the terminal vessels of the internal carotid arteries, accompanied by the subsequent formation of compensatory collateral vessels. MMD is an infrequent and progressive genetic disorder primarily observed in East Asian countries, notably Japan, with relatively low incidence and prevalence rates [2]. A comprehensive nationwide survey conducted in Japan in 1995 revealed a prevalence of 3.16 and an incidence rate of 0.35 per 100,000 population for MMD [2]. It is important to highlight that the incidence rates for this disorder exhibit considerable variations across different populations. A study analyzing the demographic characteristics of patients diagnosed with MMD who were admitted to hospitals in the United States between 2002 and 2008 reported an incidence rate of 0.13 per 100,000 population within the African-American population [3]. This finding indicates an exceptionally rare occurrence of MMD in the African-American population.

MMD typically manifests through the occurrence of ischemic strokes at a young age [5]. Additionally, individuals affected by MMD face a substantially heightened risk of experiencing subsequent ischemic strokes. A retrospective analysis of medical records encompassing 21 patients diagnosed with MMD revealed that the five-year likelihood of developing recurrent stroke following the initial event was determined to be 80.95% [6]. Furthermore, the study observed that the majority of these recurrent strokes manifested within the initial two years subsequent to the primary episode.

MMD has been documented as a potential complication associated with sickle cell disease. In a specific case study, unilateral MMD was observed in a patient’s disease progression [4]. Moreover, a retrospective analysis of medical records encompassing a cohort of 542 sickle cell disease patients revealed the occurrence of MMD in 23 individuals. These patients initially presented with symptoms such as stroke and headaches [7]. Further analysis indicated that moyamoya syndrome was observed bilaterally in 70% of the patients, while the remaining 30% exhibited unilateral manifestations.

The management of MMD primarily revolves around the prevention of further neurological decline and the effective handling of associated complications. Surgical revascularization techniques are employed as a treatment strategy to enhance blood flow to the regions of the brain experiencing inadequate perfusion due to arterial stenosis. This approach aims to alleviate ischemic symptoms and prevent subsequent cerebrovascular events. Additionally, the comprehensive management of MMD involves addressing related complications such as headaches and seizures, employing appropriate therapeutic interventions targeted at symptom relief, and optimizing patient outcomes.

## Conclusions

Patients afflicted with sickle cell disease are susceptible to a range of complications arising from the underlying pathology. One noteworthy complication that can arise is the emergence of MMD. In the presence of stroke or stroke-like symptoms and seizures occurring during the early stages of life, healthcare professionals should consider investigating MMD as a potential etiological factor. Such investigation is crucial for ensuring a timely diagnosis and enabling the implementation of revascularization procedures to avert further deterioration and mitigate the risk of cognitive impairment.
